# Influence of the fixation/permeabilization step on peptide nucleic acid fluorescence *in situ* hybridization (PNA-FISH) for the detection of bacteria

**DOI:** 10.1371/journal.pone.0196522

**Published:** 2018-05-31

**Authors:** Rui Rocha, Carina Almeida, Nuno F. Azevedo

**Affiliations:** 1 LEPABE—Laboratory for Process Engineering, Environment, Biotechnology and Energy, Department of Chemical Engineering, Faculty of Engineering of the University of Porto, Porto, Portugal; 2 CEB—Centre of Biological Engineering, University of Minho, Braga, Portugal; 3 BIOMODE—Biomolecular Determination S.A., Braga, Portugal; 4 INIAV, IP- National Institute for Agrarian and Veterinary Research, Rua dos Lagidos, Lugar da Madalena, Vairão, Vila do Conde, Portugal; Purdue University, UNITED STATES

## Abstract

Fluorescence *in situ* Hybridization (FISH) is a versatile, widespread and widely- used technique in microbiology. The first step of FISH—fixation/permeabilization—is crucial to the outcome of the method. This work aimed to systematically evaluate fixation/permeabilization protocols employing ethanol, triton X-100 and lysozyme in conjugation with paraformaldehyde for Peptide Nucleic Acid (PNA)-FISH. Response surface methodology was used to optimize these protocols for Gram-negative (*Escherichia coli* and *Pseudomonas fluorescens*) and Gram-positive species (*Listeria innocua*, *Staphylococcus epidermidis* and *Bacillus cereus*). In general, the optimal PNA-FISH fluorescent outcome in Gram-positive bacteria was obtained employing harsher permeabilization conditions when compared to Gram-negative optimal protocols. The observed differences arise from the intrinsic cell envelope properties of each species and the ability of the fixation/permeabilization compounds to effectively increase the permeability of these structures while maintaining structural integrity. Ultimately, the combination of paraformaldehyde and ethanol proved to have significantly superior performance for all tested bacteria, especially for Gram-positive species (*p*<0.05).

## Introduction

Fluorescence *in situ* Hybridization (FISH) is a widely used technique in the field of microbiology [1]. Since the first application to microorganisms by DeLong *et al*. [2], FISH progressed into a versatile technique allowing the identification, quantification and characterization of phylogenetically defined microbial populations in complex environments [3].

A standard FISH protocol targeting the rRNA, involves 4 different steps: fixation/permeabilization, hybridization, washing and visualization/detection [4,5]. The fixation/permeabilization step is crucial in FISH. On the one hand, it must preserve the integrity of rRNA, cell shape and prevent cell loss through lysis; on the other hand, it must permeabilize the cells in order to allow the diffusion of the probes through the cell envelope [1,6]. Fixation of bacteria usually employs 4% (wt/vol) paraformaldehyde, an aldehyde that cross-links cellular macromolecules, namely proteins, ultimately creating a mesh type structure within the cell [1,6,7]. The use of paraformaldehyde for most Gram-negative bacteria is sufficient to have a successful FISH outcome. However, some Gram-negative and many Gram-positive species require the use of permeabilization agents such as enzymes, solvents, detergents or even hydrochloric acid [1,5]. These will cause physical damage on the organized structure of the cell envelope in the form of pores, from where the probes can access the interior of the cell. The choice of the permeabilization procedure to be employed will depend on the characteristics of the microorganism(s) and their cell envelope composition [8], ultimately requiring a pre-optimization stage in order to assess the conditions that provide the best results [6,9,10,11].

Improvements at a procedure level, or as a result of combination with other techniques, allowed the emergence of a diverse array of FISH-based assays [12]. One example of this is the application of peptide nucleic acid (PNA) as probes. PNA is a DNA mimic composed by a neutral polyamide backbone with recognized superior hybridization features, such as improved thermal stability of the duplexes [13,14], easier diffusion through the bacterial envelope [15] and increased resistance to nucleases and proteases [3,16,17].

Even though improvements to FISH are noticeable, its outcome is still influenced by a wide variety of abiotic and biotic variables and the way they interplay with each other [18,19,20]. While biotic variation is mainly attributed to the physiological state of microorganisms, abiotic variation is mainly associated to protocol implementation, such as the type of fixative used (aldehyde or alcohol-based fixation), composition of the hybridization solution, hybridization time and temperature. Recent works have successfully disclosed the effect of temperature, time, pH, formamide, probe and dextran sulfate concentration in PNA-FISH through the application of response surface methodology (RSM) [19,21]. However, a systematic study addressing the effects of the type of fixation/permeabilization protocol in PNA-FISH is lacking.

This work aimed to disclose the effect (and interplay) of different strategies in the fixation/permeabilization step on PNA-FISH efficiency for bacteria. To this end, three different permeabilization compounds, ethanol, triton X-100 and lysozyme were combined with paraformaldehyde in a series of fixation/permeabilization protocols. Response surface methodology was then used to model the hybridization of a universal *Eubacteria* PNA probe (EUB338) [21,22] and signal quantification was assessed by flow cytometry.

## Materials and methods

### Bacterial strains

The bacterial strains used in this study were the ones selected in previous works of PNA-FISH modelling and optimization [19,21], including *Pseudomonas fluorescens* ATCC 13525, *Escherichia coli* CECT 434, *Staphylococcus epidermidis* RP61A, *Listeria innocua* CECT 910 and *Bacillus cereus*. All strains were grown on tryptic soy agar (TSA) [3% (wt/vol) tryptic soy broth and 1.5% (wt/vol) agar] (Liofilchem, Italy) at 30°C and streaked onto fresh plates every 2 or 3 days.

### PNA-FISH method

To evaluate the influence of the type of fixation/permeabilization step in the fluorescent signal outcome, a PNA-FISH protocol similar to the one described in Rocha *et al*. [19] and Santos *et al*. [21] was implemented, followed by signal quantification using flow cytometry. A universal PNA probe EUB338 (5’-TGCCTCCCGTAGGA-3’), based on the work of Amann *et al*. [22], which recognizes a conserved region of the 16S rRNA in the domain *Eubacteria*, was used. The probe was synthesized and labelled at the N terminus with AlexaFluor488 via a double 8-amino-3,6-dioxaoctanoic acid (AEEA) linker (Panagene, South Korea).

Overnight grown bacterial cells were harvested from plates and suspended in phosphate-buffered saline (PBS) (137mM NaCl [Sigma, USA]; 2.7mM KCl [Sigma]; 10mM Na_2_HPO_4_.2H_2_O [Sigma] and 1.8mM KH_2_PO_4_ [Sigma]) to a final concentration of 10^8^ to 10^9^ cells/mL. For sample fixation/permeabilization, three strategies were evaluated, in representation of different classes of permeabilizers: organic solvents (ethanol), detergents (triton X-100) and enzymes (lysozyme). The ranges selected are presented in Table 1. The conditions were selected to cover the normally used procedures described in the literature [9–11,14,23–25].

**Table 1:** Central composite design levels for the variables used to evaluate the influence of the type of fixation/permeabilization protocol in PNA-FISH. For *E*. *coli*, *P*. *fluorescens*, *L*. *innocua*, *S*. *epidermidis* and *B*. *cereus* species.

Assay	Variables		Range and level				
			−*α*	−1	0	+1	+*α*
1^a^	*x*_1_	Time in Paraformaldehyde 4% (wt/vol) (min)	9.6	30.0	60.0	90.0	110.5
	*x*_2_	[Ethanol] % (vol/vol)	8.0	25.0	50.0	75.0	92.0
	*x*_3_	Time in Ethanol (min)	4.8	15.0	30.0	45.0	55.2
1^b^	*x*_1_	Time in Paraformaldehyde 4% (wt/vol) (min)	9.6	30.0	60.0	90.0	110.5
	*x*_2_	[Triton X-100] % (vol/vol)	0.1	0.6	1.3	2.0	2.5
	*x*_3_	Time in Triton X-100 (min)	4.8	15.0	30.0	45.0	55.2
1^c^	*x*_1_	Time in Paraformaldehyde 4% (wt/vol) (min)	9.6	30.0	60.0	90.0	110.5
	*x*_2_	[Lysozyme] (mg/mL)	0.1	1.1	2.6	4.0	5.0
	*x*_3_	Time in Lysozyme (min)	4.8	15.0	30.0	45.0	55.2

^a^Experimental levels set for Paraformaldehyde-Ethanol fixation/permeabilization studies. Ethanol solutions were prepared in deionized H_2_O.

^b^Experimental levels set for Paraformaldehyde-Triton X-100 fixation/permeabilization studies. Triton X-100 solutions were prepared in deionized H_2_O.

^c^Experimental levels set for Paraformaldehyde-Lysozyme fixation/permeabilization studies. Lysozyme solutions were prepared in PBS.

One mL of previously prepared cell suspensions were pelleted by centrifugation at 10,000 × *g* for 5 min, resuspended in 400 μL of 4% (wt/vol) paraformaldehyde (Sigma) and incubated at room temperature according to the experimental design. After centrifugation at 10,000 × *g* for 5 min, the pellet was resuspended in 500 μL of ethanol (Fisher Scientific, USA), triton X-100 (Sigma) or lysozyme (from chicken egg white, ~70000 U/mg—Sigma) and incubated at -20°C, room temperature or 37°C, respectively, according to the experimental design. For hybridization, 100 μL of the previously fixed bacteria cells were pelleted by centrifugation at 10,000 × *g* for 5 min and resuspended in 100 μL of hybridization solution. The composition of the hybridization solution used took into consideration the optimum conditions already evaluated in previous studies [19,21] with the exception of probe concentration that was kept at 200 nM. Briefly, hybridization solution for *E*. *coli* and *P*. *fluorescens* contained 2% (wt/vol) dextran sulfate (average 500,000 Molecular Weight—Sigma), 0.1% (vol/vol) triton X-100 (Sigma), 5.5% (vol/vol) formamide (Sigma) and 50 mM Tris-base (pH 10; Sigma). For *L*. *innocua* and *S*. *epidermidis* it contained 10% (wt/vol) dextran sulfate, 0.1% (vol/vol) triton X-100 (Sigma), 5.5% (vol/vol) formamide (Sigma) and 50 mM Tris-HCl (pH 8; Sigma). Finally, for *B*. *cereus* the solution had the same composition as the one used for *L*. *innocua* and *S*. *epidermidis* except for the formamide concentration, which was of 49.5% (vol/vol). Samples were hybridized at 60°C for 55 min, except for *B*. *cereus* samples that were incubated for 110 min. Negative controls were prepared using the same conditions stated previously and resuspended in hybridization solution without probe. After hybridization, cells were centrifuged, at 10,000 × *g* for 5 min, resuspended in 500 μL of washing solution containing 5 mM Tris base (pH 10; Sigma), 15 mM NaCl (Sigma) and 0.1% (vol/vol) triton X-100 (Sigma) and incubated for 30 min at 60°C. After centrifugation, at 10,000 × *g* for 5 min, the pellet was resuspended in 500 μL sterile saline solution, 0.9% (wt/vol) NaCl (Sigma). Each experiment was performed in triplicate.

### Flow cytometry analysis

The fluorescence intensity of hybridized samples and negative controls was quantified by a Sony EC800 flow cytometer (Sony Biotechnology Inc., USA) equipped with a 488 nm argon ion laser. Forward angle light scatter (FS), side angle light scatter (SS) and green (FL1) fluorescence were detected at logarithmic scale. A minimum of 40,000 events falling into the bacterial gate defined on the FS-SS plot were acquired per sample at a flow rate of 20 μL/min. The data was analysed with Sony analysis software (Sony Biotechnology Inc), and the average fluorescence intensity was determined for each triplicate experiment.

### Response surface methodology (RSM)

The evaluation of the impact that each type of fixation/permeabilization step in the fluorescent signal outcome of bacteria was accessed recurring to RSM, accordingly to the procedure applied by Santos *et al*. [21]. The average fluorescence intensity obtained after PNA-FISH was used as the dependent variable.

Central composite designs (CCD) were set up for *P*. *fluorescens*, *E*. *coli*, *S*. *epidermidis*, *L*. *innocua* and *B*. *cereus* using the statistical software Design Expert^®^ 10.0.5.0 (Stat-Ease Inc., USA) to estimate the coefficients of the model. The range and levels of all variables were defined according to previous studies [9–11,14,23–25]. Each CCD included 2^3^ factorial points (coded at ± 1), 6 axial points (coded as ± α) that represent extreme values used for the estimation of the model curvature and 6 centre points (all factors at coded level 0) repeated to take into account the experimental error [26,27]. Therefore, each design matrix consisted of 20 PNA-FISH experiments.

### Statistical analysis

In order to infer the best fixation/permeabilization procedure for all five species, the average fluorescence intensity values obtained by flow cytometry were introduced in Design Expert^®^ 10.0.5.0 software to fit a quadratic model and each obtained model was analysed using analysis of variance (ANOVA). The interaction of the three independent variables and their effect on the fluorescence intensity was analysed by constructing the response surface and contour plots. The optimization function of the software was then used to estimate the optimum conditions within the experimental range that maximized the fluorescence intensity.

A confirmation experiment of the predicted optimum points for the 3 fixation/permeabilization protocols was performed simultaneously for each species in triplicate. The fluorescence intensity obtained in the confirmation experiments was evaluated using a one-way ANOVA followed by Tukey’s test to assess the significance between the different fixation/permeabilization protocols for each species. The ANOVA and Tukey’s test analysis were performed in the software GraphPad Prism 5 (GraphPad Software, Inc., USA).

## Results and discussion

This work intended to study and model the effect of different fixation/permeabilization strategies of bacteria during a PNA-FISH procedure. This step is of the utmost importance in FISH, since it can dictate the success or failure of the whole procedure. To model its effect, RSM was applied to the hybridization data obtained from 3 Gram-positive (*S*. *epidermidis*, *L*. *innocua* and *B*. *cereus*) and 2 Gram-negative species (*P*. *fluorescens* and *E*. *coli*). These species were selected in order to include bacteria with different cell wall thicknesses (from thin, *e*.*g*. Gram-negative *P*. *fluorescens* [2.41 ± 0.54 nm, values for *P*. *aeruginosa* excluding the outer membrane], to thick cell walls, *e*.*g*. Gram-positive *B*. *cereus* [33.3 ± 4.7 nm, values for *B*. *subtillis*] [28]) and as a follow up of previous modelling and optimization works [19,21].

Initial CCD designs were based on the values typically described in the literature for FISH fixation/permeabilization protocols [9–11,14,23–25]. It should be noticed that paraformaldehyde at a concentration of 4% (wt/vol) is a common step to most of the procedures (as this is a preferential compound for fixative purposes) and the main procedural differences are related to the type of permeabilization agent used, as well as the concentration and exposure periods [1,6]. The application of the fixation/permeabilization protocols according to the CCD designs and their application to PNA-FISH for the five different species under study was successful, since significant quadratic models (for at least one of the test conditions at each fixation/permeabilization combination) (*p*-value <0.05) and satisfactory coefficients of determination (*R*^*2*^) were obtained (Tables A and B in S2 File). This allowed to determine the optimal conditions for maximum fluorescence (Table 2).

**Table 2:** Optimum PNA-FISH fixation/permeabilization protocols predicted through RSM models for each species. Fixation/permeabilization combinations included: paraformaldehyde and ethanol, paraformaldehyde and triton X-100 and paraformaldehyde and lysozyme. Negative control, predicted and average obtained fluorescence with error values (standard deviation) under optimum conditions are presented.

Bacteria	Fixation/Permeabilization Protocol	Optimum conditions			Predicted Fluorescence (a.u.)	Confirmation Experiments	
		Time in Paraformaldehyde 4% (wt/vol) (min)	[Permeabilization Agent] % (vol/vol) or (mg/mL)	Time in Permeabilization Agent (min)		Obtained Fluorescence (a.u.)	Negative Control (a.u.)
*P*. *fluorescens*	Paraformaldehyde + Ethanol	53.1	25.0	15	215.3	370 ± 30	7.6
	Paraformaldehyde + Triton X-100	70.0	2.0	15	344.0	420 ± 70	16.0
	Paraformaldehyde + Lysozyme	90.0	1.1	15	348.5	350 ± 40	8.7
*E*. *coli*	Paraformaldehyde + Ethanol	89.9	25.1	15	205.8	290 ± 10^a^	11.4
	Paraformaldehyde + Triton X-100	82.9	2.0	15	179.3	278 ± 4^a^	11.7
	Paraformaldehyde + Lysozyme	90.0	1.1	15	160.6	151 ± 7	8.9
*S*. *epidermidis*	Paraformaldehyde + Ethanol	30.0	51.3	15	105.2	102 ± 1^b^	8.8
	Paraformaldehyde + Triton X-100	90.0	2.0	45	67.2	75.2 ± 0.6^b^	7.5
	Paraformaldehyde + Lysozyme	90.0	4.0	15	28.9	38 ± 2^b^	7.5
*L*. *innocua*	Paraformaldehyde + Ethanol	30.0	25.0	45	126.5	160 ± 10	12.6
	Paraformaldehyde + Triton X-100	35.2	2.0	45	146.4	210 ± 30	7.5
	Paraformaldehyde + Lysozyme	90.0	1.5	45	163.4	180 ± 40	7.6
*B*. *cereus*	Paraformaldehyde + Ethanol	90.0	75.0	15	2136.8	1700 ± 200^c^	21.9
	Paraformaldehyde + Triton X-100	88.8	0.6	15	1861.0	1000 ± 300	21.6
	Paraformaldehyde + Lysozyme	86.0	1.1	15	1062.7	1200 ± 100	18.0

^a^Indicates significant differences among the fixation/permeabilization protocol and the one using lysozyme, *p*<0.05

^b^Indicates significant differences among all fixation/permeabilization protocols, *p*<0.05

^c^Indicates significant differences among the fixation/permeabilization protocol using ethanol and the ones using triton X-100 and lysozyme, *p*<0.05.

**Fig 1:**
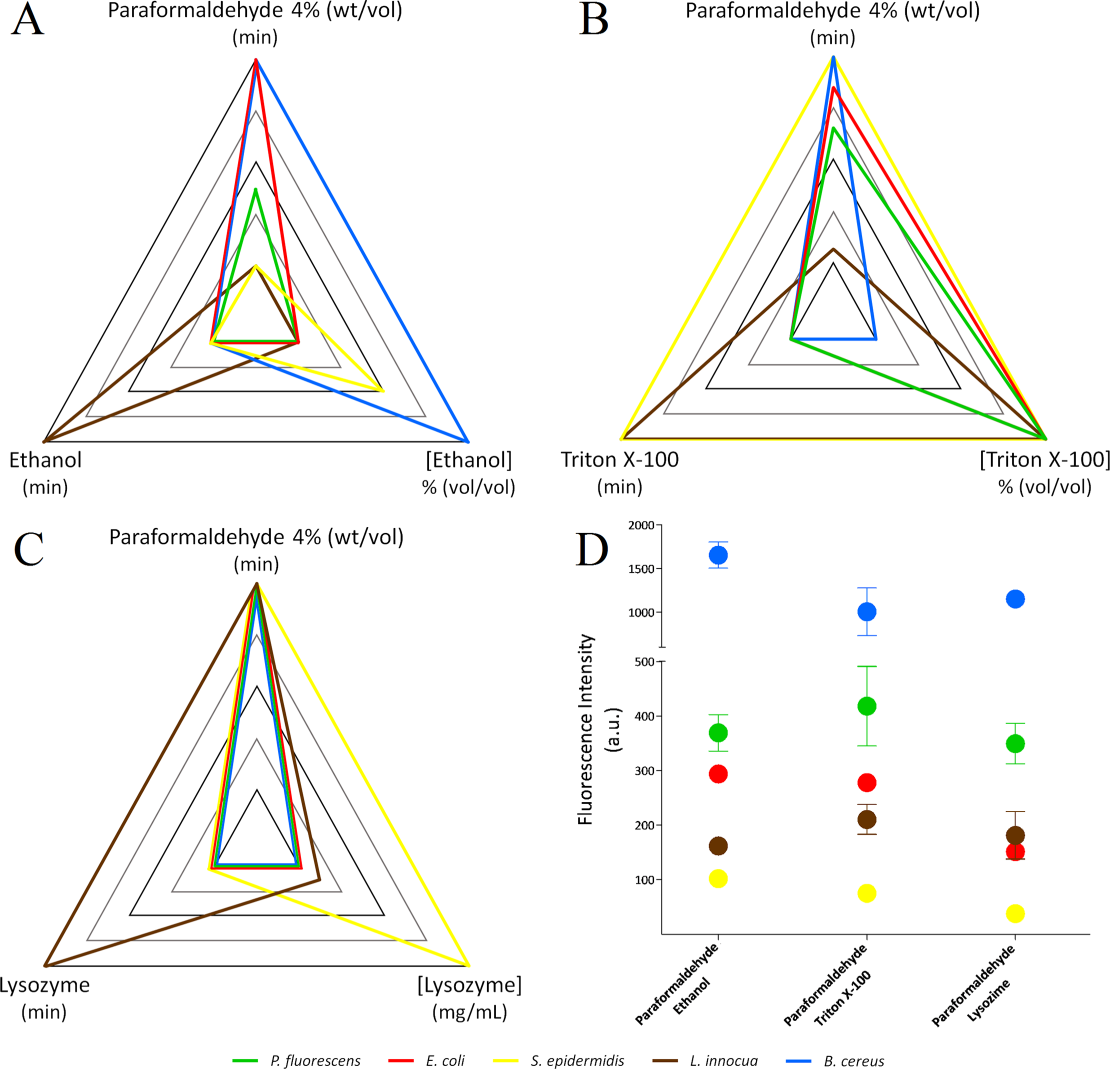
Optimum PNA-FISH fixation/permeabilization protocol and fluorescence intensity outcome obtained for each species. Radar chart representation in terms of level factors (-1 [inner vertices] to 1 [outer vertices]): A—Paraformaldehyde and ethanol; B—Paraformaldehyde and triton X-100; C—Paraformaldehyde and lysozyme. D—Average fluorescence intensity and error bars (standard deviation) of the confirmation experiment for the optimum fixation/permeabilization protocol for each species (*P*. *fluorescens*—green; *E*. *coli*—red; *S*. *epidermidis*—yellow; *L*. *innocua*—brown and *B*. *cereus*—blue).

As in previous optimization studies, differences in fluorescence intensity values between species are observed. The fluorescence signal for positive samples ranged from 17.4 to 2449.0 a.u., depending on the microorganism and condition tested. Overall, higher values were obtained for Gram-negative over Gram-positive species, except for *B*. *cereus*. These variations are known to be, at a certain level, intrinsic to the target RNA content and probe accessibility [19,21]. As significant variations in the fluorescence intensity can be found between species when applying different fixation/permeabilization protocols for each bacteria (see Table 2 and Fig 1D).

For each fixation/permeabilization method, the results in Table 2 were transposed to level factors and plotted into a radar chart for further analysis (Fig 1).

### Treatment with paraformaldehyde and ethanol

Ethanol is used in FISH procedure as a fixative and as a permeabilization agent [7,11,14]. Ethanol fixative capability, similarly to other alcohols, arises from the coagulation, precipitation and denaturation of proteins, through the interference with their hydration cloud [7,29]. On the other hand, permeabilization is accomplished by promoting the solubilization of cell envelope components [8].

Analyzing Fig 1A, a Gram-specific behavior is observed in the optimal fixation/permeabilization protocol found for each species. Gram-negative *P*. *fluorescens* and *E*. *coli* required longer paraformaldehyde steps (above 50 minutes) combined with low ethanol concentrations for short periods (25% [vol/vol] for 15 minutes). These findings are not surprising, since previous reports using DNA probes, stated that hybridization of Gram-negative species can be successfully achieved using only paraformaldehyde as a fixation/permeabilization agent [5,10,11]. This arises from the fact that aldehyde fixatives present also a weak detergent-like activity [5]. On the other side, Gram-positive *S*. *epidermidis* and *B*. *cereus* required exposure to higher ethanol concentrations (50% and 75% [vol/vol], respectively) or, as found for *L*. *innocua*, a longer ethanol step (45 minutes at 25% [vol/vol]) for an effective permeabilization. Again, this was an anticipated result since Gram-positive bacteria are known to be harder to permeabilize [10]. Overall, these optimizations are directly connected with the specific cell envelope architecture, while Gram-positive species cell wall is mainly composed of thick and rigid peptidoglycan structure intertwined with teichoic and lipoteichoic acids, Gram-negative species present a small layer of peptidoglycan between the cell membrane and an outer membrane [30–32].

Furthermore, it is also possible to observed that short paraformaldehyde steps of 30 minutes, are preferred for Gram-positive species, excluding *B*. *cereus*. These results are in line with previous reports stating detrimental effects of cross-linking agents in terms of fluorescent outcome on whole cell hybridization of Gram-positive species [33,34].

### Treatment with paraformaldehyde and triton X-100

Triton X-100 is a nonionic chemical surfactant used in FISH in the fixation/permeabilization step or/and as part of the hybridization solution [23–25,35]. It is a very effective detergent in the solubilization of phospholipids, due to the high binding affinity to hydrophobic molecules. Permeabilization arises from a channel-forming effect that results from two main events: interaction and substitution of cell envelope lipid molecules and conformational changes in cell envelope proteins [8,36].

Analyzing paraformaldehyde and triton X-100 testing results, Fig 1B, is possible to observe a Gram-specific pattern, as with previous paraformaldehyde/ethanol optimizations. Overall, Gram-negative species, *P*. *fluorescens* and *E*. *coli*, required less exposure to triton X-100 (15 minutes) in order to achieve the highest fluorescence intensity than Gram-positive species, *L*. *innocua* and *S*. *epidermidis* (45 minutes).

An interesting finding regarding *B*. *cereus* optimal protocol was encountered, since the optimal triton X-100 concentration and exposure time were considerably lower (0.6% [vol/vol] for 15 minutes) than those found for the other Gram-positive species. This could result from the use of a relatively high formamide content in the hybridization solution (resulting from previous optimizations—49.5% [vol/vol]), which is known to have a damaging effect on the integrity of the cell wall and thus might present a synergetic effect with triton X-100 treatment [21].

### Treatment with paraformaldehyde and lysozyme

Lysozyme is a lytic enzyme that hydrolyses the β-1,4 glycosidic bonds between N-acetylglucosamine and N-acetylmuramic acid of peptidoglycan [37]. Since peptidoglycan is a common component of the cell wall of *Eubacteria*, especially in Gram-positive species, this enzyme is typically used for permeabilization of bacteria in FISH procedures [9,11,23,37]. As with other lytic enzymes, lysozyme has a narrow applicability spectrum when compared to chemical permeabilization. This results from the specificity of the enzyme-target reaction and loss of activity if somehow their action site is inaccessible and/or modified [37].

Analyzing paraformaldehyde and lysozyme results (Fig 1C), a Gram-specific pattern is observed again. Gram-negative species present a higher fluorescent outcome with a fixation/permeabilization step with long exposures to paraformaldehyde (90 minutes) and short exposures to lysozyme (15 minutes) at low concentrations (1.1 mg/mL). Generally, in Gram-negative species the outer membrane precludes the access to lytic enzymes; thus, membrane removal by detergents or chelating agents is usually required for a successful permeabilization [38]. However, the compromised membranes of fixed cells assure the enzyme access to the peptidoglycan. In fact, an extended exposure to lysozyme could result on cell lysis even before Gram-positive cells became permeable [9,37]. The results obtained here seem to confirm this last observation, since higher exposure to lysozyme would induce a lower PNA-FISH fluorescence outcome in Gram-negative bacteria, likely due to extensive damage in the cell envelope.

In Gram-positive species, *B*. *cereus* presents a behavior similar to the one observed for Gram-negative species. The optimal protocol for *L*. *innocua* required an higher lysozyme exposure (45 minutes), while *S*. *epidermidis* required an higher lysozyme concentration (4.0 mg/mL). This species-specific behavior could be related to lysozyme sensitivity/resistance of each species, the degree of cross-linking, type and content of glycan modifications in the peptidoglycan, which are characteristics that can affect lysozyme activity [39–44]. One clear example of this is the observed low fluorescence outcome of *S*. *epidermidis* (Table 2). In fact, the *Staphylococcus* genus is known to have a peptidoglycan insensitive to lysozyme activity, resulting mainly from modifications (O-acetylation) of peptidoglycan monomers [39]. As such, *S*. *epidermidis*, was expected to be poorly permeabilized by paraformaldehyde/lysozyme protocols.

### Towards a fully optimized PNA-FISH procedure

Following fixation/permeabilization protocol optimization for each species, the predictions made by the different models were confirmed experimentally. From the confirmation experiments, a general agreement between the predicted and the obtained fluorescence values was observed (Table 2). Furthermore, these assays also enable a comparison between the different fixation/permeabilization protocols (Table 2 and Fig 1D). For *B*. *cereus* the fixation/permeabilization protocol using ethanol performed significantly better than the other two tested (*p*<0.05). Regarding *E*. *coli* and S. epidermidis both ethanol and triton X-100 protocols worked significantly better than the one using lysozyme (*p*<0.05). For *P*. *fluorescens* and *L*. *innocua* all tested fixation/permeabilization protocols provided similar PNA-FISH outcomes (*p*>0.05). Based on the above, paraformaldehyde and ethanol was the fixation/permeabilization PNA-FISH protocol which allowed an overall higher fluorescence outcome in all five *Eubacteria* species tested.

The results obtained here can be combined with previous optimizations and subsequently be used for the development of new PNA-FISH methodologies for microbial detection [19,21]. In fact, putting all this information together, an almost fully optimized PNA-FISH procedure can be obtained in accordance to the properties of the target bacteria (Table 3).

**Table 3:** Optimized methodological variables for PNA-FISH. Conditions for 5 Gram-positive and Gram-negative species, obtained by RSM in this work and in previous studies.

Variable		Fixation/Permeabilization			Hybridization					
		Paraformaldehyde 4% (wt/vol) (min)	Ethanol % (vol/vol)	Ethanol (min)	Time (minutes)	Temperature (°C)	Formamide (% vol/vol)	pH	DS % (wt/vol)	Probe (nM)
Bacteria	*P*. *fluorescens*	50	25	15	55	60	5.5	10	2	≥ 300
	*E*. *coli*	90	25	15	55		5.5	10	2	
	*L*. *innocua*	30	25	45	55		5.5	8	10	
	*S*. *epidermidis*	30	50	15	55		5.5	8	10	
	*B*. *cereus*	90	75	15	120		49.5	8	10	

Although the results point towards a species-specific optimal fixation/permeabilization protocol, a compromise between protocol and fluorescence outcome in PNA-FISH is possible. This arises from the observation that highly fluorescent species, such *B*. *cereus* and *P*. *fluorescens*, will exhibit a similar or higher fluorescence outcome when compared other low fluorescent-species, such *S*. *epidermidis*, *E*. *coli* and *L*. *innocua*, even when protocol conditions are very different from their optimal procedures (Figures A, B and C in S1 File). These observations have particular significance in multiplex applications where several species can be targeted in a single assay [5,45]. Namely, the species with lower rRNA content (thus weaker basal fluorescence signal) might be favored in terms of hybridization and permeabilization protocols, so the population’s signals can be balanced.

Finally, it is possible that the optimized conditions for the fixation/permeabilization protocols can be applicable to other microorganisms when PNA probes are used. Nonetheless, adjustments to the optimum conditions described in this work cannot be excluded, especially for target species with very different cell envelope compositions. It is also important to notice that the optimizations described above are likely not applicable to DNA, RNA and other nucleic acid mimics probes such as LNA or 2’OMe RNA, as their molecular structure differs markedly from PNA oligonucleotides [4].

## Conclusions

**Fig 2:**
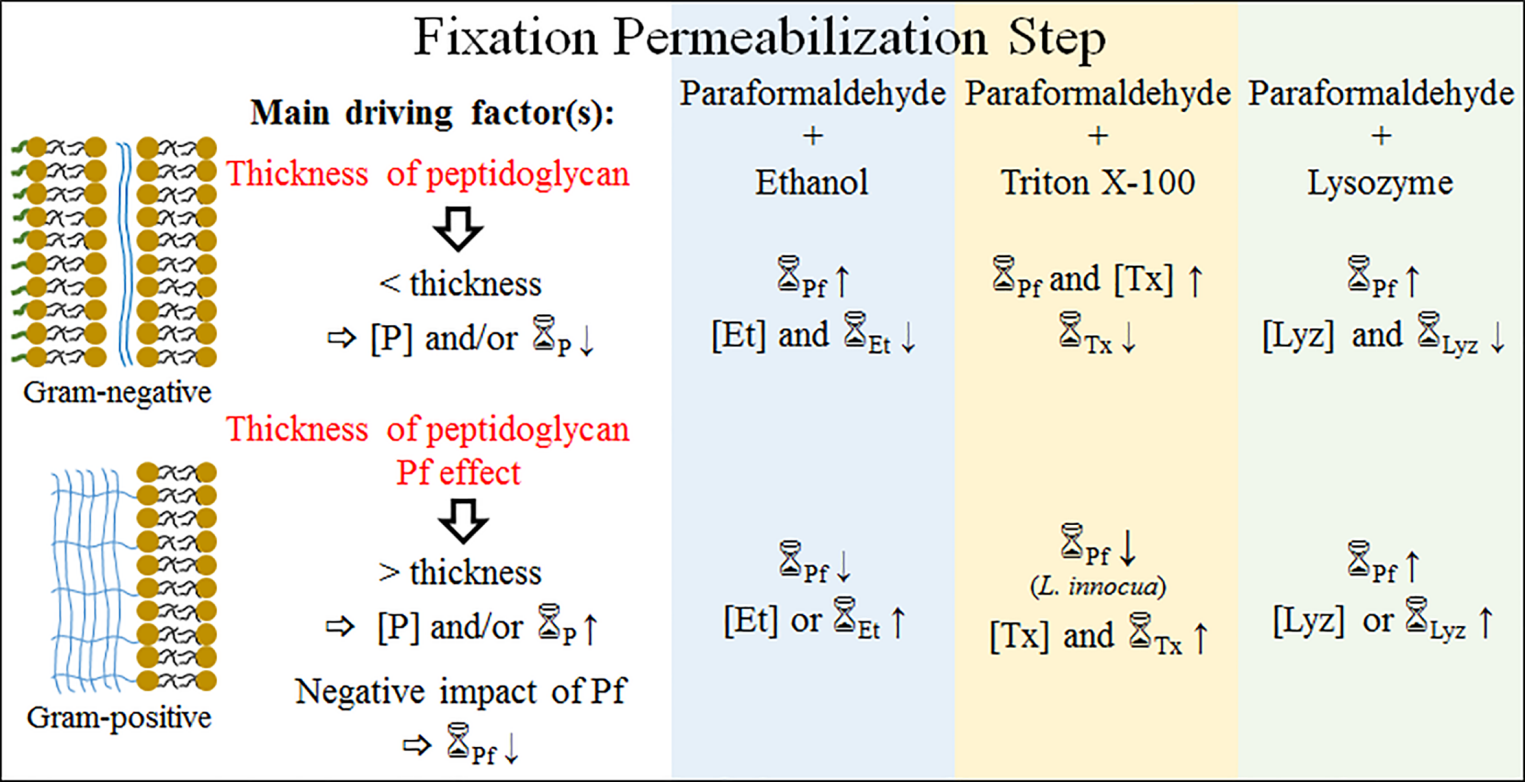
Identification of the driving factors that influence each fixation/permeabilization protocol in PNA-FISH for Gram-positive and Gram-negative species (except *B*. *cereus*). P—Permeabilizant; Pf—Paraformaldehyde; Et—Ethanol; Tx—Triton X-100; Lyz—Lysozyme; [Xx]—Concentration of permeabilizant X; _Xx_—Duration of substance X application.

In this work we have shown that paraformaldehyde fixation followed by organic solvent (ethanol), detergent (triton X-100) or enzymatic (lysozyme) permeabilization are suitable strategies for PNA-FISH procedures targeting *Eubacteria*. However, a unique optimal protocol was not found for all tested species. Despite this, of the three tested strategies, paraformaldehyde followed by ethanol has proven to be the best fixation/permeabilization protocol for PNA-FISH procedures. The differences between optimal protocols obtained were mainly attributed to the inherent differences in the cell envelope, more precisely in terms of peptidoglycan thickness. As such, for Gram-negative species with a thinner peptidoglycan cell wall structure, the combination of a short step with low concentration of permeabilizant provided the best PNA-FISH outcomes. On the contrary, Gram-positive species with a thicker peptidoglycan cell wall structure, a longer step and/or higher permeabilizant concentrations were required for an optimal PNA-FISH outcome (Fig 2). Additionally, the duration of the paraformaldehyde step was identified as another driving factor for Gram-positive species, especially for ethanol procedures. Prolonged exposure proved to have a detrimental effect on the fluorescence outcome and as such, short procedures were generally preferred.

Further research, could focus in the expansion of the scope of this optimization to a broader range permeabilization compounds, microorganisms, including species from the other two Domains, *Archaea* and *Eukarya*, and eventually, to a set of different nucleic acid mimic probes.

## Supporting information

S1 File Surface response plots for the fluorescence response of the five tested bacteria regarding each fixation/permeabilization protocol tested. (DOCX)

S2 File Adjusted quadratic models and analysis of variance (ANOVA) parameters obtained for all tested bacteria according to the fixation/permeabilization protocol tested. (DOCX)

S3 File Confirmations data file. (XLSX)
