# Correction: Influence of the fixation/permeabilization step on peptide nucleic acid fluorescence *in situ* hybridization (PNA-FISH) for the detection of bacteria

**DOI:** 10.1371/journal.pone.0208867

**Published:** 2018-12-05

**Authors:** Rui Rocha, Carina Almeida, Nuno F. Azevedo

In the Funding section, the reference for the funder Center of Biological Engineering is listed incorrectly. The correct reference is: UID/BIO/04469.
