# Supplementary material for: Influence of the fixation/permeabilization step on peptide nucleic acid fluorescence in situ hybridization (PNA-FISH) for the detection of bacteria
Source: PLoS One. 2018 May 31;13(5):e0196522. doi: 10.1371/journal.pone.0196522 (PMC5979007; doi:10.1371/journal.pone.0196522)
Supplement: S1 File — (DOCX) [file pone.0196522.s001.docx]

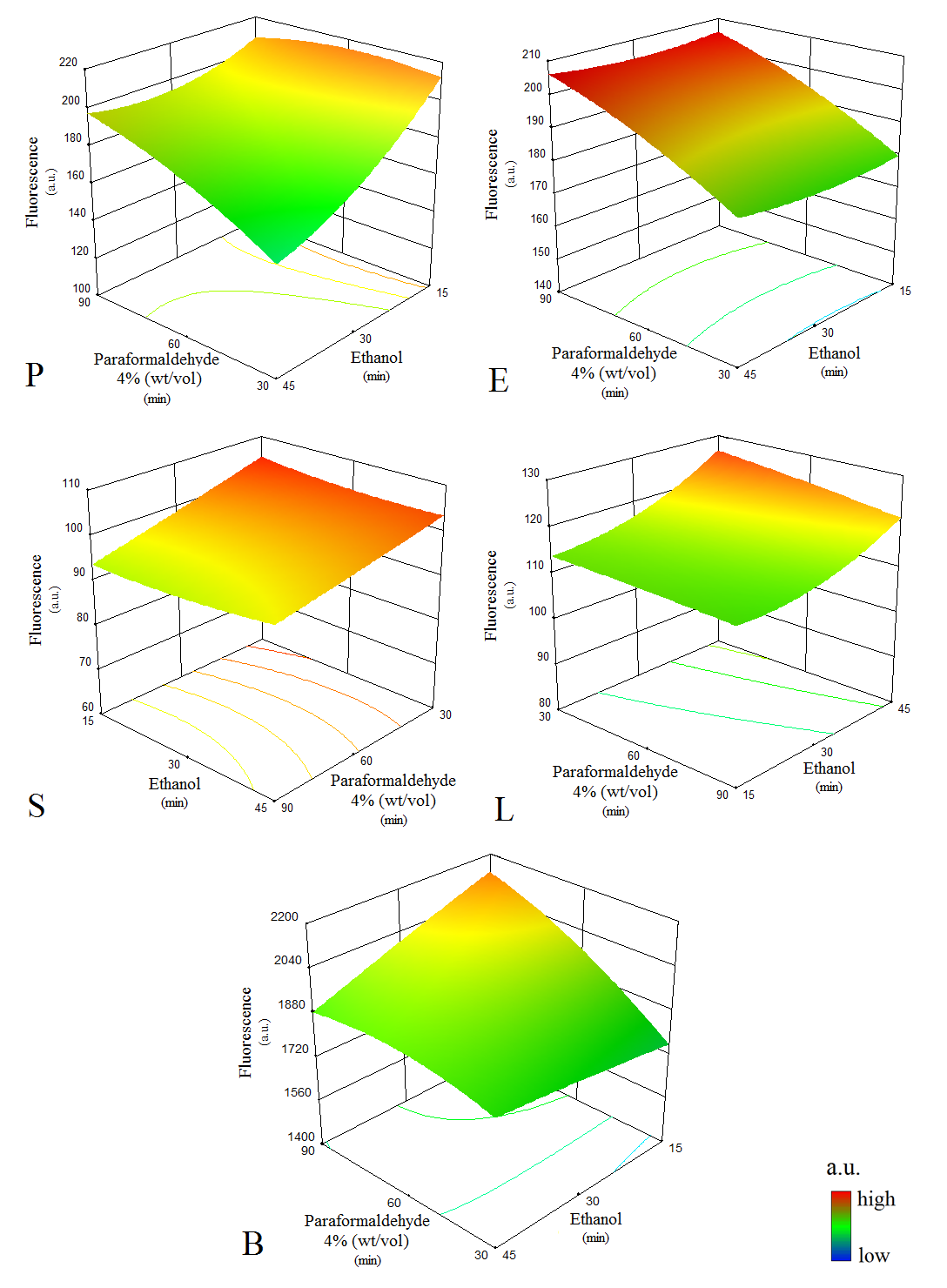


**Figure A** - Surface response plots for the fluorescence response of *P. fluorescens* (P), *E. coli* (E), *S. epidermidis* (S), *L. innocua* (L) and *B. cereus* (B), regarding the fixation/permeabilization protocol using paraformaldehyde and ethanol. The permeabilizant concentration was kept at their optimum value in each graph. Fluorescence values are presented in arbitrary units (a.u.).


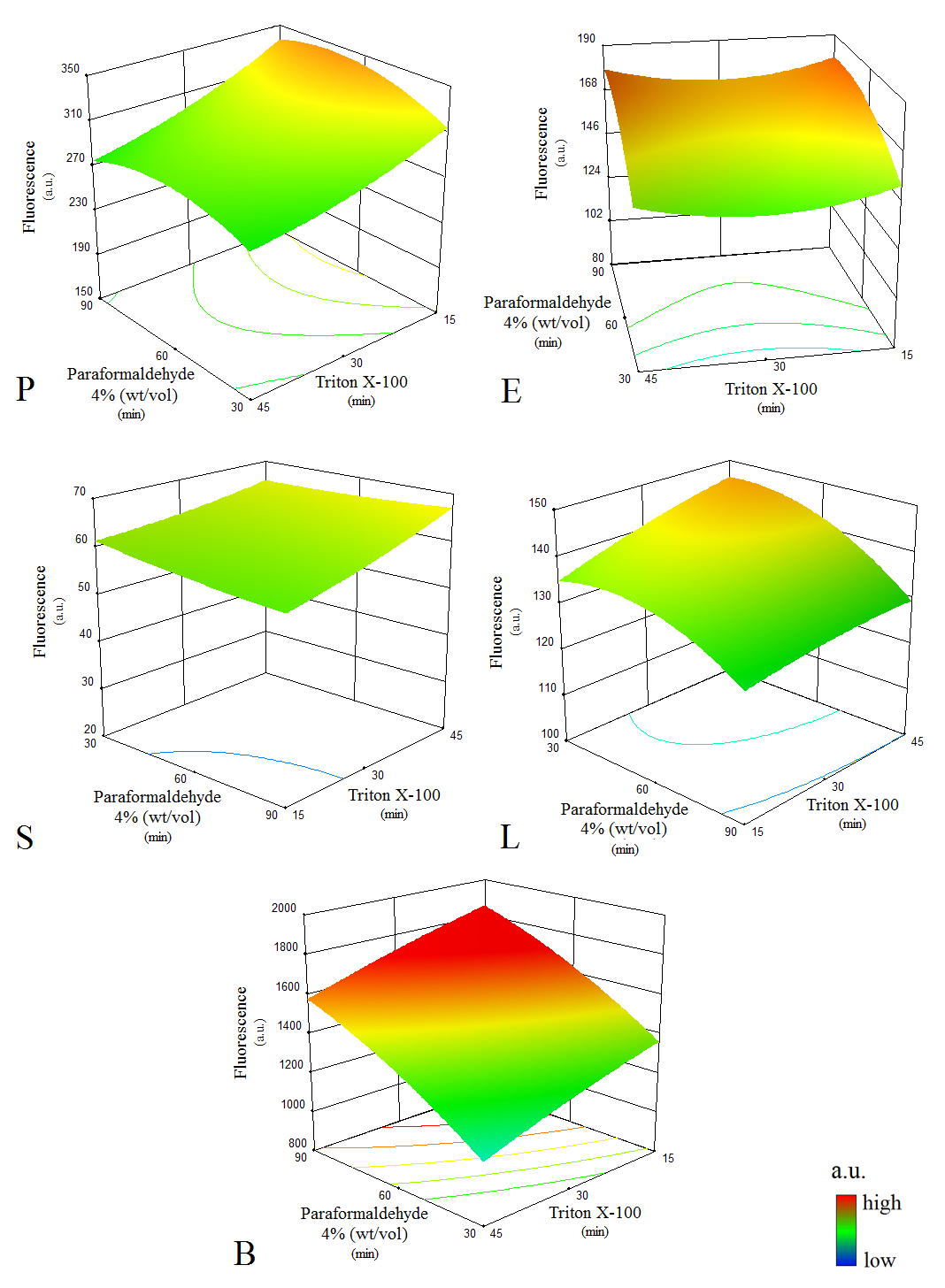


**Figure B** - Surface response plots for the fluorescence response of *P. fluorescens* (P), *E. coli* (E), *S. epidermidis* (S), *L. innocua* (L) and *B. cereus* (B), regarding the fixation/permeabilization protocol using paraformaldehyde and triton X-100. The permeabilizant concentration was kept at their optimum value in each graph. Fluorescence values are presented in arbitrary units (a.u.).


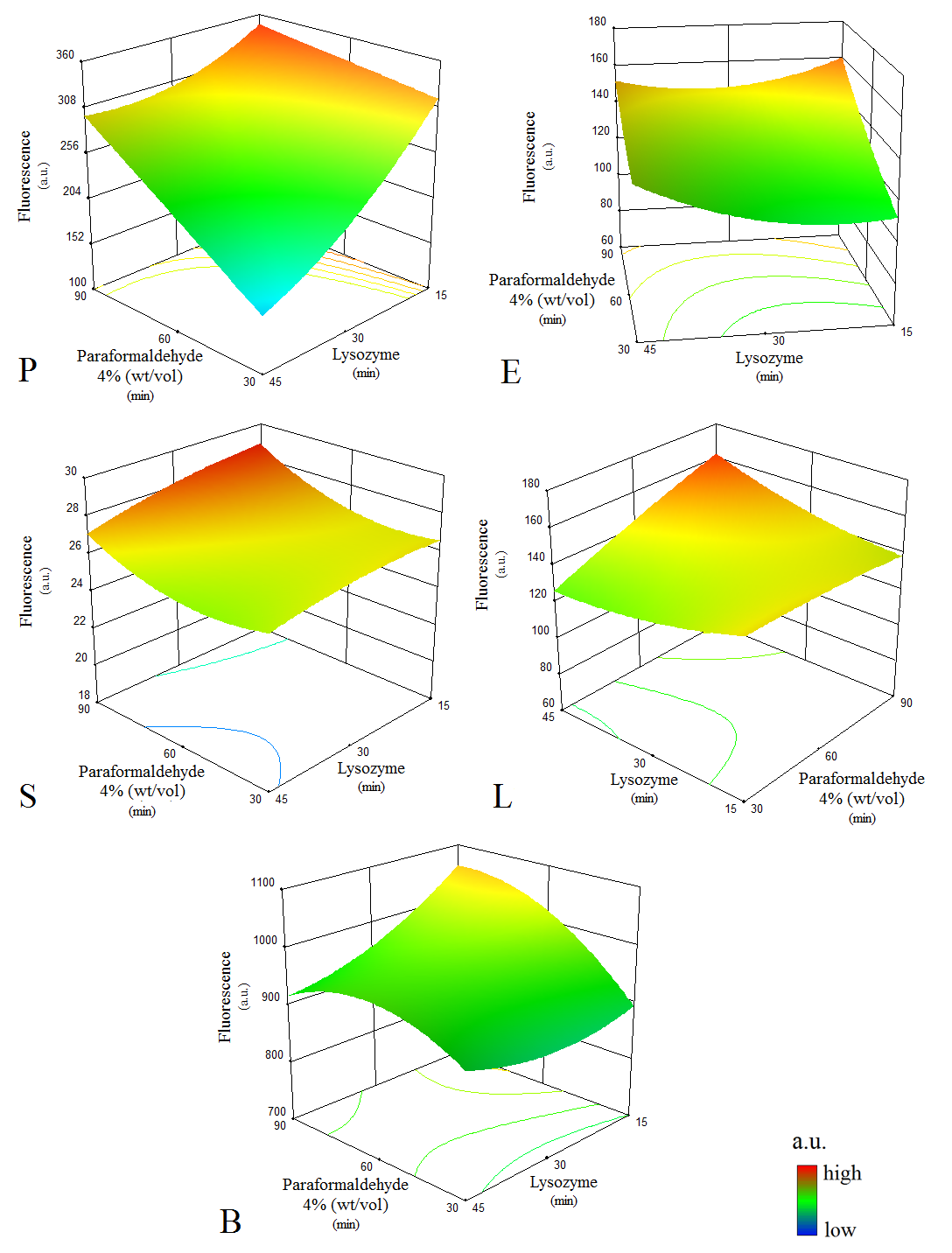


**Figure C -** Surface response plots for the fluorescence response of *P. fluorescens* (P), *E. coli* (E), *S. epidermidis* (S), *L. innocua* (L) and *B. cereus* (B), regarding the fixation/permeabilization protocol using paraformaldehyde and lysozyme. The permeabilizant concentration was kept at their optimum value in each graph. Fluorescence values are presented in arbitrary units (a.u.).
