# Supplementary material for: Influence of the fixation/permeabilization step on peptide nucleic acid fluorescence in situ hybridization (PNA-FISH) for the detection of bacteria
Source: PLoS One. 2018 May 31;13(5):e0196522. doi: 10.1371/journal.pone.0196522 (PMC5979007; doi:10.1371/journal.pone.0196522)
Supplement: S2 File — (DOCX) [file pone.0196522.s002.docx]

**Table A.** Adjusted quadratic models for the different bacteria in study, in terms of coded values, considering the effect of time in Paraformaldehyde 4% (wt/vol) (min) ($x_{1}$), [Permeabilizant] (% vol/vol for Ethanol and Triton X-100 and mg/mL for Lysozyme) ($x_{2}$) and time in Permeabilizant (min) ($x_{3}$) and their interactions on the predicted fluorescence intensity (Y).

| Species | Fixation/Permabilization Protocol | Model |
| --- | --- | --- |
| *P. fluorescens* | Paraformaldehyde + Ethanol | $\frac{1}{\sqrt{\text{Y}}}=175.72+10.11x_{1}-10.71x_{2}-16.17x_{3}+1.08x_{1}x_{2}+11.19x_{1}x_{3}+0.16x_{2}x_{3}-4.67x_{1}^{2}+4.30x_{2}^{2}+7.96x_{3}^{2}$ |
|  | Paraformaldehyde + Triton X-100 | $\frac{1}{\sqrt{\text{Y}}}=282.80+19.50x_{1}+19.84x_{2}-25.58x_{3}-13.50x_{1}x_{2}-4.75x_{1}x_{3}+0.23x_{2}x_{3}-16.14x_{1}^{2}+7.62x_{2}^{2}+6.64x_{3}^{2}$ |
|  | Paraformaldehyde + Lysozyme | $\frac{1}{\sqrt{\text{Y}}}=259.42+44.55x_{1}+0.55x_{2}-36.547x_{3}+2.91x_{1}x_{2}+26.99x_{1}x_{3}+14.09x_{2}x_{3}-0.32x_{1}^{2}+6.19x_{2}^{2}+18.43x_{3}^{2}$ |
| *E. coli* | Paraformaldehyde + Ethanol | $\frac{1}{\sqrt{\text{Y}}}=180.64+9.15x_{1}-13.56x_{2}+0.84x_{3}-3.28x_{1}x_{2}-0.26x_{1}x_{3}+0.40x_{2}x_{3}-2.26x_{1}^{2}-0.092x_{2}^{2}+1.82x_{3}^{2}$ |
|  | Paraformaldehyde + Triton X-100 | $\frac{1}{\sqrt{\text{Y}}}=148.67+12.62x_{1}+18.66x_{2}+2.48x_{3}+0.084x_{1}x_{2}+0.30x_{1}x_{3}-2.74x_{2}x_{3}-8.12x_{1}^{2}-1.19x_{2}^{2}+8.17x_{3}^{2}$ |
|  | Paraformaldehyde + Lysozyme | $\frac{1}{\sqrt{\text{Y}}}=117.78+15.82x_{1}-2.63x_{2}+4.07x_{3}-0.17x_{1}x_{2}-7.66x_{1}x_{3}+0.55x_{2}x_{3}+2.68x_{1}^{2}+7.71x_{2}^{2}+9.72x_{3}^{2}$ |
| *S. epidermidis* | Paraformaldehyde + Ethanol | $\frac{1}{\sqrt{\text{Y}}}=98.95-4.45x_{1}+0.274x_{2}+0.17x_{3}-1.80x_{1}x_{2}+0.99x_{1}x_{3}+1.18x_{2}x_{3}+0.13x_{1}^{2}-8.74x_{2}^{2}+0.82x_{3}^{2}$ |
|  | Paraformaldehyde + Triton X-100 | $\frac{1}{\sqrt{\text{Y}}}=33.46-1.59x_{1}+20.09x_{2}+2.07x_{3}+0.87x_{1}x_{2}+1.44x_{1}x_{3}+1.48x_{2}x_{3}+0.45x_{1}^{2}+8.24x_{2}^{2}+0.68x_{3}^{2}$ |
|  | Paraformaldehyde + Lysozyme | $\frac{1}{\sqrt{\text{Y}}}=25.35-0.69x_{1}+2.16x_{2}+1.04x_{3}+1.57x_{1}x_{2}-0.24x_{1}x_{3}-1.66x_{2}x_{3}+0.94x_{1}^{2}-1.00x_{2}^{2}-0.294x_{3}^{2}$ |
| *L. innocua* | Paraformaldehyde + Ethanol | $\frac{1}{\sqrt{\text{Y}}}=109.77-4.94x_{1}-3.98x_{2}+2.47x_{3}-3.22x_{1}x_{2}-0.94x_{1}x_{3}-2.75x_{2}x_{3}-0.23x_{1}^{2}+2.26x_{2}^{2}+2.85x_{3}^{2}$ |
|  | Paraformaldehyde + Triton X-100 | $\frac{1}{\sqrt{\text{Y}}}=130.18-7.86x_{1}+4.62x_{2}-1.16x_{3}+1.73x_{1}x_{2}-2.02x_{1}x_{3}+4.66x_{2}x_{3}-4.95x_{1}^{2}+5.68x_{2}^{2}-0.89x_{3}^{2}$ |
|  | Paraformaldehyde + Lysozyme | $\frac{1}{\sqrt{\text{Y}}}=136.76+3.16x_{1}-16.33x_{2}+3.66x_{3}-6.29x_{1}x_{2}+10.31x_{1}x_{3}+3.24x_{2}x_{3}-1.39x_{1}^{2}-13.81x_{2}^{2}+4.12x_{3}^{2}$ |
| *B. cereus* | Paraformaldehyde + Ethanol | $\frac{1}{\sqrt{\text{Y}}}=1943.81 +68.79x_{1}+107.27x_{2}+18.24x_{3}+33.09x_{1}x_{2}-82.37x_{1}x_{3}-57.43x_{2}x_{3}-40.38x_{1}^{2} -92.62x_{2}^{2} -4.67x_{3}^{2}$ |
|  | Paraformaldehyde + Triton X-100 | $\frac{1}{\sqrt{\text{Y}}}=1549.49+113.40x_{1}-120.44x_{2}-80.70x_{3}-128.74x_{1}x_{2}-1.83x_{1}x_{3}+54.94x_{2}x_{3}-43.57x_{1}^{2}-131.56x_{2}^{2}-13.35x_{3}^{2}$ |
|  | Paraformaldehyde + Lysozyme | $\frac{1}{\sqrt{\text{Y}}}=922.97+32.62x_{1}-36.45x_{2}-71.42x_{3}-10.92x_{1}x_{2}-38.32x_{1}x_{3}-38.93x_{2}x_{3}-47.23x_{1}^{2}+8.78x_{2}^{2}+26.51x_{3}^{2}$ |

**Table B.** Analysis of variance (ANOVA) for each second-order model and each individual factor ($x_{1}$ - Time in Paraformaldehyde 4% [wt/vol] [min]; $x_{2}$ - [Permeabilizant] [% vol/vol for Ethanol and Triton X-100 and mg/mL for Lysozyme]; $x_{3}$ - Time in Permeabilizant [min]).

|  | *P. fluorescens* | | | *E. coli* | | | *S. epidermidis* | | | *L. innocua* | | | *B. cereus* | | |
| --- | --- | --- | --- | --- | --- | --- | --- | --- | --- | --- | --- | --- | --- | --- | --- |
|  | Pf + Et | Pf + Tx | Pf + Lyz | Pf + Et | Pf + Tx | Pf + Lyz | Pf + Et | Pf + Tx | Pf + Lyz | Pf + Et | Pf + Tx | Pf + Lyz | Pf + Et | Pf + Tx | Pf + Lyz |
| Model F-value | 1.98 | 1.71 | 2.62 | 4.97 | 4.52 | 1.60 | 3.60 | 9.89 | 6.09 | 1.82 | 10.70 | 15.22 | 3.51 | 5.31 | 3.89 |
| Model *p*-value | 0.1507 | 0.2076 | 0.0750 | 0.0098 | 0.0136 | 0.2372 | 0.0292 | 0.0007 | 0.0046 | 0.1810 | 0.0005 | 0.0001 | 0.0317 | 0.0077 | 0.0227 |
| Lack-of-fit F-value | 2.03 | 2.82 | 1.58 | 2.00 | 17.10 | 8.47 | 2.03 | 1.24 | 1.43 | 3.61 | 0.28 | 1.49 | 1.68 | 3.54 | 1.95 |
| Lack-of-fit *p*-value | 0.2273 | 0.1397 | 0.3145 | 0.2318 | 0.0037 | 0.0175 | 0.2282 | 0.4100 | 0.3518 | 0.0924 | 0.9060 | 0.3358 | 0.2914 | 0.0958 | 0.2408 |
| Model R^2^ | 0.6407 | 0.6061 | 0.7020 | 0.8174 | 0.8028 | 0.5901 | 0.7642 | 0.8990 | 0.8457 | 0.6216 | 0.9059 | 0.9320 | 0.7595 | 0.8271 | 0.7780 |
| $x_{1}$ F-value | 2.73 | 2.96 | 10.99 | 13.17 | 9.61 | 7.87 | 5.97 | 0.46 | 2.23 | 5.68 | 35.29 | 2.23 | 4.49 | 9.52 | 2.91 |
| $x_{1}$p-value | 0.1296 | 0.1158 | 0.0078 | 0.0046 | 0.0113 | 0.0186 | 0.0347 | 0.5119 | 0.1663 | 0.0384 | 0.0001 | 0.1666 | 0.0601 | 0.0115 | 0.1190 |
| $x_{2}$ F-value | 3.06 | 3.07 | 0.0017 | 28.92 | 21.01 | 0.22 | 0.022 | 74.05 | 22.08 | 3.69 | 12.17 | 59.39 | 10.92 | 10.74 | 3.63 |
| $x_{2}$p-value | 0.1107 | 0.1102 | 0.9684 | 0.0003 | 0.0010 | 0.6510 | 0.8862 | < 0.0001 | 0.0008 | 0.0838 | 0.0058 | < 0.0001 | 0.0079 | 0.0083 | 0.0858 |
| $x_{3}$ F-value | 6.98 | 5.10 | 7.40 | 0.11 | 0.37 | 0.52 | 0.0085 | 0.79 | 5.07 | 1.42 | 0.77 | 2.98 | 0.32 | 4.82 | 13.94 |
| $x_{3}$ p-value | 0.0247 | 0.0474 | 0.0216 | 0.7451 | 0.5561 | 0.4865 | 0.9284 | 0.3961 | 0.0480 | 0.2613 | 0.3999 | 0.1151 | 0.5864 | 0.0529 | 0.0039 |
| $x_{1}x_{2}$ F-value | 0.018 | 0.83 | 0.028 | 0.99 | 0.0002 | 0.0005 | 0.57 | 0.082 | 6.80 | 1.41 | 1.00 | 5.17 | 0.61 | 7.19 | 0.19 |
| $x_{1}x_{2}$ p-value | 0.8955 | 0.3828 | 0.8715 | 0.3429 | 0.9876 | 0.9818 | 0.4667 | 0.7803 | 0.0261 | 0.2627 | 0.3403 | 0.0464 | 0.4533 | 0.0231 | 0.6713 |
| $x_{1}x_{3}$ F-value | 1.96 | 0.10 | 2.36 | 0.0063 | 0.0031 | 1.08 | 0.17 | 0.22 | 0.16 | 0.12 | 1.37 | 13.85 | 3.77 | 0.0014 | 2.35 |
| $x_{1}x_{3}$ p-value | 0.1921 | 0.7549 | 0.1553 | 0.9066 | 0.9568 | 0.3230 | 0.6851 | 0.6466 | 0.6937 | 0.7352 | 0.2691 | 0.0040 | 0.0807 | 0.9704 | 0.1562 |
| $x_{2}x_{3}$ F-value | 0.0004 | 0.0002 | 0.64 | 0.014 | 0.27 | 0.0056 | 0.24 | 0.23 | 7.64 | 1.03 | 7.26 | 1.37 | 1.83 | 1.31 | 2.43 |
| $x_{2}x_{3}$ p-value | 0.9845 | 0.9880 | 0.4408 | 0.3796 | 0.6171 | 0.9419 | 0.6319 | 0.6384 | 0.0200 | 0.3348 | 0.0225 | 0.2685 | 0.2054 | 0.2793 | 0.1504 |
| $x_{1}^{2}$ F-value | 0.61 | 2.14 | 0.0006 | 0.85 | 4.19 | 0.24 | 0.0052 | 0.039 | 4.42 | 0.013 | 14.75 | 0.46 | 1.63 | 1.48 | 6.43 |
| $x_{1}^{2}$ p-value | 0.4515 | 0.1738 | 0.9809 | 0.3796 | 0.0678 | 0.6353 | 0.9538 | 0.8465 | 0.0619 | 0.9103 | 0.0033 | 0.5146 | 0.2301 | 0.2513 | 0.0296 |
| $x_{2}^{2}$ F-value | 0.52 | 0.48 | 0.22 | 0.0014 | 0.090 | 1.97 | 24.28 | 13.16 | 4.99 | 1.25 | 19.39 | 44.77 | 8.59 | 13.52 | 0.22 |
| $x_{2}^{2}$ p-value | 0.4872 | 0.5053 | 0.6463 | 0.9708 | 0.7707 | 0.1905 | 0.0006 | 0.0046 | 0.0495 | 0.2888 | 0.0013 | < 0.0001 | 0.0150 | 0.0043 | 0.6473 |
| $x_{3}^{2}$ F-value | 1.78 | 0.36 | 1.98 | 0.55 | 4.25 | 3.13 | 0.21 | 0.089 | 0.41 | 1.99 | 0.48 | 4.00 | 0.022 | 0.14 | 2.03 |
| $x_{3}^{2}$ p-value | 0.2113 | 0.5605 | 0.1893 | 0.4746 | 0.0662 | 0.1071 | 0.6552 | 0.7711 | 0.5383 | 0.1883 | 0.5062 | 0.0735 | 0.8854 | 0.7169 | 0.1850 |

Pf - Paraformaldehyde; Et - Ethanol; Tx - Triton X-100; Lyz - Lysozyme.
